# 1107. Vancomycin AUC Dosing: Is One Concentration in the Hand Worth Two in the Bush?

**DOI:** 10.1093/ofid/ofab466.1301

**Published:** 2021-12-04

**Authors:** Justin Spivey, Jenny Shroba, Connor Deri, Cara Nys, Rebekah Wrenn, Michael E Yarrington

**Affiliations:** 1 Duke University Medical Center, Durham, North Carolina; 2 Duke Raleigh Hospital, Raleigh, North Carolina; 3 Duke University Hospital, Durham, North Carolina; 4 Duke University, Durham, North Carolina; 5 Duke Center for Antimicrobial Stewardship and Infection Prevention, Durham, North Carolina

## Abstract

**Background:**

Recent guidelines recommend a transition from trough-based to area-under the curve-based (AUC) monitoring for vancomycin for serious invasive methicillin-resistant *Staphylococcus aureus* infections. Due to the challenges of performing AUC monitoring in clinical practice, this study sought to compare the accuracy of an AUC calculated from two points using trapezoidal calculations and from a single steady-state trough combined with population assumptions.

**Methods:**

This prospective cohort analysis included hospitalized patients with stable renal function from 10.2020 to 12.2020 with two vancomycin concentrations obtained at steady-state during a single dosing interval. For each patient, AUC was calculated via trapezoidal equations utilizing peak and trough concentrations (P/T) and using the trough concentration (T) combined with population volume of distribution. Appropriate concentrations were defined as a peak at least 2 hours after the end of the infusion and a trough within one hour of the next dose. The percent and actual differences were calculated between the P/T and T AUC assessments for each patient. A patient level review was independently conducted by two clinical pharmacists to evaluate if a change in dosing would have been made according to AUC estimation methodology.

**Results:**

Thirty-one patients had appropriate steady-state P/T obtained. Baseline demographics are shown in Table 1 with the majority of patients being overweight with normal renal function. The mean calculated AUC for both groups was similar, P/T 544.8 and T 549.8. The mean and median percent differences were 1.85% and 0.65%, with a standard deviation of 7.3% and an apparent normal distribution (Figure 1, p = 0.94 by Shapiro’s test). The median absolute difference in AUC was 25.82 mg*h/L between methodologies. Both methods would have resulted in the same modification to the vancomycin regimen based on patient level chart review.

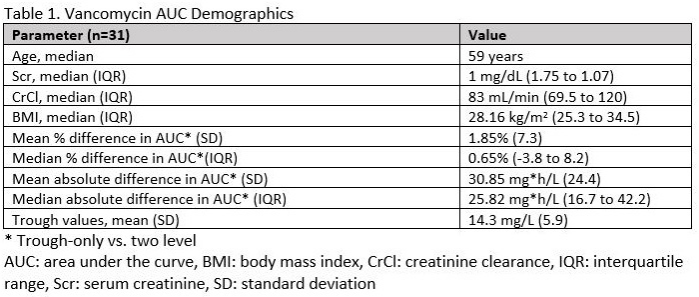

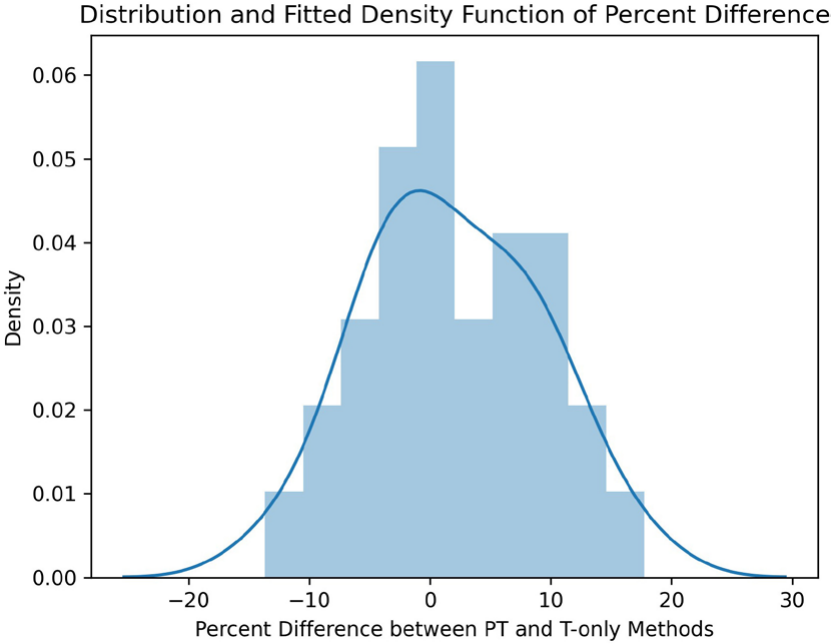

**Conclusion:**

The single-trough method performed similarly to the more laborious P/T method. No patient would have received a dose adjustment based on the two different AUC estimation methods. The single-trough method may represent a resource and workflow conscious AUC estimation method for patients meeting population assumptions.

**Disclosures:**

**All Authors**: No reported disclosures

